# Ophthalmoscopic Assessment of the Retinal Nerve Fiber Layer. The Beijing Eye Study

**DOI:** 10.1371/journal.pone.0062022

**Published:** 2013-04-29

**Authors:** Yaqin Zhang, Liang Xu, Li Zhang, Hua Yang, Ya Xing Wang, Jost B. Jonas

**Affiliations:** 1 Beijing Institute of Ophthalmology, Beijing Tongren Hospital, Capital Medical University, Beijing, China; 2 Department of Ophthalmology, Medical Faculty Mannheim of the Ruprecht-Karls-University, Heidelberg, Germany; Univeristy of Melbourne, Australia

## Abstract

**Purpose:**

To examine the retinal nerve fiber layer (RNFL) ophthalmoscopically, to search for localized RNFL defects, and to assess factors associated with RNFL visibility in a population-based setting.

**Methods:**

The population-based cross-sectional Beijing Eye Study 2006 included 3251 subjects. Using color fundus photographs, RNFL visibility was assessed in grades from 0 to 8 in 8 fundus sectors. Localized RNFL defects were defined as wedge-shaped defects running towards the optic disc.

**Results:**

After exclusion of subjects with optic media opacities, 2602 subjects (mean age:58.1±9.0 years) were included. RNFL visibility score was highest (*P*<0.001) in the temporal inferior region, followed by the temporal superior region, nasal superior region, and nasal inferior region. In multivariate analysis, higher RNFL visibility score was associated with younger age (*P*<0.001;standardized coefficient beta:−0.44;regression coefficient B: −0.22; 95%CI: −0.24, −0.20), female gender (*P*<0.001;beta:0.11;B:1.00;95%CI:0.67,1.32), higher blood concentration of low-density lipoproteins (*P* = 0.002;beta:0.07;B:0.34;95%CI:0.13,0.56), absence of dyslipidemia (*P* = 0.001;beta: −0.07;B: −0.58;95%CI: −0.93, −0.24), lower blood glucose concentration (*P* = 0.006;beta: −0.05;B: −0.14;95%CI: −0.24, −0.04), hyperopic refractive error (*P*<0.001;beta:0.15;B:0.45;95%CI:0.34,0.56), smaller optic disc size (*P*<0.001;beta: −0.08; B:−0.72;95% CI:−1.04, −0.40), absence of glaucomatous optic neuropathy (*P*<0.001;beta: −0.06;B: −2.69;95%CI:–4.18, −1.21) and absence of non-glaucomatous optic nerve damage (*P* = 0.001;beta: −0.06;B: −4.80;95%CI:0. −7.64, −1.96). Localized RNFL defects were detected in 96 subjects (prevalence:3.7±0.45% (95% confidence interval(CI):3.0,4.4). In multivariate analysis, prevalence of localized RNFL defects was associated with higher blood pressure (*P*<0.001; odds ratio (OR):1.07;95%CI:1.03,1.10), higher concentration of low-density lipoproteins (*P* = 0.01;OR:1.42;95%CI:1.08,1.85), higher prevalence of glaucomatous optic neuropathy (*P*<0.001;OR:46.8;95%CI:19.4,113) and diabetic retinopathy (*P* = 0.002;OR:3.20;95%CI:1.53,6.67), and lower total RNFL visibility (*P*<0.001;OR:0.92;95%CI:0.88,0.96).

**Conclusions:**

In Chinese aged 45+ years, a decreased RNFL visibility was associated with older age, male gender, dyslipidemia, hyperglycemia, myopia, larger optic disc, and glaucomatous or non-glaucomatous optic neuropathy. Localized RNFL defects (prevalence:3.7±0.45%) were correlated mainly with higher blood pressure, higher concentration of low-density lipoproteins, glaucomatous optic neuropathy and diabetic retinopathy. These data are helpful for the routine ophthalmoscopic examination of the RNFL.

## Introduction

The retinal nerve fiber layer (RNFL) is part of the afferent visual pathway and contains the retinal ganglion cell axons. Any optic nerve damage affects the RNFL through the loss of nerve fibers. The RNFL loss occurs in a diffuse and/or in a localized pattern [1]–[3]. Localized RNFL defects appear as wedge-shaped dark areas running towards, but not necessarily touching, the optic disc border. The defects follow an arcuate pattern according to the normal course of the retinal nerve fibers from their retinal ganglion cell bodies to the optic nerve head [2], [3]. The importance of localized defects of the retinal nerve fiber layer for the diagnosis of glaucoma and other optic nerve disease has been demonstrated in many previous studies [3]–[7]. The evaluation of the RNFL has become an integral part of the routine ophthalmoscopic examination. Previous studies have suggested that localized RNFL defects occur in about 20% of all glaucoma eyes, but that they can also be found in other diseases of the optic nerve and retina, such as optic disk drusen, toxoplasmotic retinochoroidal scars, longstanding papilledema, diabetic retinopathy and arterial hypertensive retinopathy [8]. The special diagnostic importance of RNFL assessment is based on the findings that a localized RNFL defect is not found in a normal eye, that particularly in glaucomatous eyes with small optic discs, a localized RNFL defect can point to a glaucomatous optic nerve damage even if the optic disc appears to be normal; and that in non-glaucomatous optic nerve damage the shape of the neuroretinal rim inside of the optic disc remains mostly unchanged while the RNFL can show localized RNFL defects or a diffuse RNFL loss [7].

In view of the clinical importance of the assessment of the RNFL, we examined the RNFL on conventional color fundus photographs as surrogate of a normal ophthalmoscopic examination, graded the visibility of the RNFL in 8 fundus sectors, searched for localized RNFL defects, and looked for factors and diseases associated with the overall visibility of the RNFL and with the occurrence of localized RNFL defects. We examined a population-based study sample to avoid the confounding effects of a bias due to a referral effect potentially adherent to any hospital-based study. Knowledge about the parameters examined could further enhance the diagnostic utility of the clinical RNFL assessment. The findings could also be compared with the results of other imaging techniques, such as spectral-domain optical coherence tomography, to visualize and examine the RNFL.

## Methods

### Ethics Statement

The Medical Ethics Committee of the Beijing Tongren Hospital approved the study protocol and all participants gave informed written consent, according to the Declaration of Helsinki.

The Beijing Eye Study is a population-based study, which was performed in 2001 and included 4439 subjects out of 5324 subjects invited to participate with an age of 40+ years (response rate: 83.4%). The study has been described in detail previously [9], [10]. In 2006, all study subjects were invited to be re-examined. Out of the 4439 subjects, 3251 (73.2%) returned for the re-examination, while 143 (3.2%) had died and 1045 (23.5%) refused to participate or had moved away. The survey of 2006 was divided into the rural part (1,500 (46.1%) subjects) and the urban part (1,751 (53.9%) subjects). The mean age was 60.4±10.0 years (range: 45–89 years). For the present study, only data measured in the survey of 2006 were taken.

All examinations were carried out in the communities, either in schoolhouses or in community houses. An ophthalmic examination was carried out including measurement of uncorrected and best corrected visual acuity, non-contact tonometry, frequency doubling perimetry, slit lamp-based biomicroscopy ophthalmoscopy, and photography of the cornea, lens (Neitz CT-R camera; Neitz Instruments Co., Tokyo, Japan) and fundus with the pupils medically dilated (fundus camera, type CR6-45 NM, Canon Inc. USA). Glaucoma was defined according to the criteria of the International Society of Geographic and Epidemiological Ophthalmology ISGEO [11]. Non-glaucomatous optic nerve damage was defined as increased pallor of the neuroretinal rim, decreased diameter of the retinal arteries, and a normally shaped neuroretinal rim according to the inferior-superior-nasal-inferior (ISNT) rule [12]. The minimum criterion for diagnosis of diabetic retinopathy was the presence of at least one microaneurysm. Using the optic disc photographs, the diameters of the arteries and veins of the four main retinal vessel trunks were measured at the optic disc border. Smaller branches of the retinal vessels were not taken into account. The raw data of the retinal vessel diameter measurements were corrected for the magnification by the optic media of the eye and the fundus camera using Littmanńs method as described above. The technique has already been described in detail [13]. Besides an ophthalmologic examination, the past history of ocular and systemic diseases was assessed in a questionnaire. The concentrations of glucose, cholesterol, low-density lipoproteins, and high-density lipoproteins were determined in fasting blood samples. The blood pressure was measured with the participant sitting for at least 5 min. Arterial hypertension was defined as a systolic blood pressure ≥140 mm Hg and/or a diastolic blood pressure ≥90 mm Hg, and/or self-reported current treatment for arterial hypertension with antihypertensive medication. Diabetes was defined as fasting glucose concentrations ≥7.0 mmol/L and self-reported diagnosis of diabetes. Dyslipidemia was defined as any of hypercholesterolemia (total cholesterol concentration ≥5.72 mmol/L (220 mg/dL)) or hypertriglyceridemia (triglyceride concentration ≥1.70 mmol/L (150 mg/dL)) or low high-density lipoprotein-cholesterol (HDL-C concentration ≤0.91 mmol/L (35 mg/dL)) or a positive history for dyslipidemia. For the statistical analyses, the total prevalence of dyslipidemia (abnormal blood examinations results and/or positive history for dyslipidemia) was taken.

The RNFL was assessed on the fundus photographs and the photographs of the optic disc. A localized RNFL defect was defined as a wedge shaped and not spindle-like defect, running towards or touching the optic disc border for not more than 60 degrees of the optic disc circumference. The visibility of the retinal nerve fiber layer was examined in eight sectors centered around the optic disc: the temporal inferior sector, the temporal superior sector, the nasal superior sector, and the nasal inferior sector, each sector along one of the four major vessel bundles; and in four additional sectors located between the four major vessel bundles: the temporal horizontal sector, the superior sector, the nasal sector, and the inferior sector. For each sector, the visibility of the retinal nerve fiber layer was graded using a score ranging between “0” for “no visibility” to “8” for “very good visibility”. The photographs were assessed by an experienced ophthalmologist (YQZ) in a masked manner without knowledge of the history, morphometric optic disc data, perimetric results, and other data. In case of doubt, the photographs were re-examined by a panel including several ophthalmologists (YQZ, YXW, LX, JBJ). The techniques has already been described in detail previously [13].

Statistical analysis was performed using a commercially available statistical software package (SPSS for Windows, version 20.0, IBM-SPSS, Chicago, IL). The results of only one eye per subject were taken for statistical analysis. In a first step, we examined the mean values (presented as mean ± standard deviation). Frequencies were presented as mean ± standard error. In a second step, we performed univariate analyses with the RNFL visibility score or the presence of localized RNFL defects as dependent parameters and ocular and general parameters as independent parameters. In a third step, we carried out multivariate analyses, with the RNFL visibility score or the presence of localized RNFL defects as dependent parameters and all those parameters as independent parameters which were significantly associated with the RNFL visibility score or the presence of localized RNFL defects in univariate analysis. We dropped step by step those parameters from the list of independent variables which no longer showed a significant association with the RNFL variables, starting with the parameter with the highest *P*-value. Confidence intervals of a range of 95% (95%CI) were presented. All *P*-values were 2-sided and were considered statistically significant when the values were less than 0.05.

## Results

Retinal fundus photographs were available for 3097 (95.3%) subjects. Due to reasons such advanced cataract, vitreous opacities and a very bright fundus, usually in highly myopic eyes, the detectability of the RNFL was so profoundly reduced that additional 495 participants were excluded from the study, which eventually included 2602 subjects with a mean age of 58.1±9.0 years (median: 57 years; range: 45–86 years) and a mean refractive error of −0.03±1.51 diopters (median: +0.25 diopter; range: −12.88 to +7.25 diopters).

Mean total RNFL visibility score was 27.8±4.5 (median: 28; range: 8–68). It was significantly (*P*<0.001) the highest in the temporal inferior region (5.1±1.0), followed by the temporal superior region (4,8±0.7), the nasal superior region (3.9±1.0), the nasal inferior region (3.5±0.8), the superior region (3.2±0.7), the inferior region (2.8±0.7), the temporal region (2.6±0.6) and finally the nasal region (2.0±0.5).

The mean total RNFL visibility score was significantly associated with younger age (*P*<0.001). After adjustment for age, higher RNFL visibility score was significantly correlated with the systemic parameters of female gender (*P*<0.001), rural region of habitation (*P*<0.001), lower body height (*P<*0.001), lower body weight (*P* = 0.02), lower level of education (*P* = 0.002), higher fasting blood concentration of low-density lipoproteins (*P* = 0.006), lower prevalence of dyslipidemia (*P* = 0.046), lower fasting blood concentration of glucose (*P* = 0.001) and lower prevalence of ever smoking (*P* = 0.001); and with the ocular parameters of hyperopic refractive error (*P*<0.001), lower intraocular pressure (*P* = 0.03), smaller optic disc area (*P* = 0.001), smaller parapapillary alpha zone (*P* = 0.04) and smaller parapapillary beta zone (*P*<0.001), lower presence of glaucomatous optic neuropathy (*P*<0.001), lower prevalence of non-glaucomatous optic nerve damage (*P* = 0.001), lower presence of diabetic retinopathy (*P* = 0.02), lower presence of optic disc hemorrhages (*P* = 0.01), lower prevalence of localized RNFL defects (*P*<0.001), and higher best corrected visual acuity (*P*<0.001). After adjusting for age, RNFL visibility score was not significantly associated with the systemic parameters of body mass index (*P* = 0.39), systolic blood pressure (*P* = 0.17), diastolic blood pressure (*P* = 0.13), mean arterial blood pressure (*P* = 0.12), prevalence of arterial hypertension (*P* = 0.94), fasting blood concentration of cholesterol (*P* = 0.23), blood concentration of high-density lipoproteins (*P* = 0.06), prevalence of diabetes mellitus (*P* = 0.06), presence of early age-related macular degeneration (*P* = 0.97) and ocular perfusion pressure (*P* = 0.53), nor with the ocular parameters of neuroretinal rim area (*P* = 0.86).

The multivariate analysis included the RNFL visibility score as dependent variable and as independent variables all parameters which were significantly associated with the RNFL visibility score in univariate analysis. After step-wise dropping of intraocular pressure (*P* = 0.57), body height (*P* = 0.46), presence of diabetic retinopathy (*P* = 0.87), level of education (*P* = 0.51), ever smoking (*P* = 0.26), presence of optic disc hemorrhages (*P* = 0.34), body weight (*P* = 0.11), region of habitation (*P* = 0.07), and parapapillary alpha zone (*P* = 0.06), a higher RNFL visibility score remained to be significantly associated with younger age (*P*<0.001), female gender (*P*<0.001), higher blood concentration of low-density lipoproteins (*P* = 0.002), absence dyslipidemia (*P* = 0.001), lower blood concentration of glucose (*P* = 0.006), hyperopic refractive error (*P*<0.001), smaller optic disc size (*P*<0.001), absence of glaucomatous optic neuropathy (*P*<0.001) and absence of non-glaucomatous optic nerve damage (*P* = 0.001) (Table 1). If area of parapapillary beta zone was added as independent parameter, smaller beta zone was highly significantly (*P*<0.001; standardized coefficient beta: −0.12; regression coefficient: −0.81 (95%CI): −1.06, −0.56) associated with a higher RNFL visibility score, while the association between a higher RNFL visibility score and absence of glaucoma became marginally significant (*P* = 0.049).

**Table 1:** Associations (Multivariate Analysis) between the Retinal Nerve Fiber Layer Visibility Score and Ocular and systemic Parameters in the Beijing Eye Study 2006.

Parameter	*P*-Value	StandardizedCoefficient Beta	RegressionCoefficient	95% ConfidenceInterval
Age (Years	<0.001	−0.44	−0.22	−0.24, −0.20
Gender (Men/Women)	<0.001	0.11	1.00	0.67, 1.32
Blood Concentration Low-Density Lipoproteins (mmol/L)	0.002	0.07	0.34	0.13, 0.56
Presence of Dyslipidemia	0.001	−0.07	−0.58	−0.93, −0.24
Fasting Blood Glucose Concentration (mmol/L)	0.006	−0.05	−0.14	−0.24, −0.04
Refractive Error (Diopters)	<0.001	0.15	0.45	0.34, 0.56
Optic Disc Area (mm2)	<0.001	−0.08	−0.72	−1.04, −0.40
Presence of Glaucomatous Optic Neuropathy	<0.001	−0.06	−2.69	−4.18, −1.21
Presence of Non-Glaucomatous Optic Neuropathy	0.001	−0.06	−4.80	−7.64, −1.96

A localized RNFL defect was detected in 96 subjects with a prevalence of 3.7±0.45% (95%CI: 3.0, 4.4). In univariate analysis, the prevalence of localized RNFL defects was significantly associated with older age (*P* = 0.009; OR: 1.03 (95%CI: 1.01, 1.05)). After adjustment for age, a higher prevalence of localized RNFL defects was significantly correlated with the systemic parameters of rural region of habitation (*P* = 0.01), higher body mass index (*P* = 0.047), higher systolic blood pressure (*P*<0.001), higher diastolic blood pressure (*P* = 0.001), higher mean arterial blood pressure (*P*<0.001), higher prevalence of arterial hypertension (*P*<0.001), higher fasting blood concentration of cholesterol (*P* = 0.03), low-density lipoproteins (*P* = 0.02) and glucose (*P* = 0.001), and higher prevalence of diabetes mellitus (*P*<0.001); and with the ocular parameters of smaller neuroretinal rim area (*P*<0.001), lower total RNFL visibility (*P*<0.001), presence of glaucomatous optic neuropathy (*P*<0.001), presence of optic disc hemorrhages (*P* = 0.003), higher ocular perfusion pressure (*P* = 0.001), presence of non-glaucomatous optic nerve damage (*P* = 0.02), presence of diabetic retinopathy (*P*<0.001), presence of retinal vein occlusions (*P*<0.001), presence of early age-related macular degeneration (*P* = 0.02), and lower best corrected visual acuity (*P* = 0.002). After adjusting for age, presence of localized RNFL defects was not significantly associated with the systemic parameters of gender (*P* = 0.96), body height (*P* = 0.49), body weight (*P* = 0.25), level of education (*P* = 0.18), blood concentration of high-density lipoproteins (*P* = 0.89), presence of dyslipidemia 07) and ever smoking (*P* = 0.28); nor with the ocular parameters of refractive error (*P* = 0.14), intraocular pressure (*P* = 0.12), optic disc area (*P* = 0.37), and area of parapapillary alpha zone (*P* = 0.92) and parapapillary beta zone (*P* = 0.81).

In a first step of the multivariate analysis, we added all systemic parameters which were significantly associated with localized RNFL defects in the univariate analysis, to the list of independent parameters and removed step by step those parameters which the highest *P*-value. After dropping body mass index (*P* = 0.99), blood cholesterol concentration (*P* = 0.94), systolic blood pressure (*P* = 0.56), region of habitation (*P* = 0.41) and diastolic blood pressure (*P* = 0.28) from the list, higher age (*P* = 0.01) and higher blood concentrations of glucose (*P* = 0.004) and low-density lipoproteins (*P* = 0.06) remained as parameters with a *P*-value of less than 0.10. In a second step of the multivariate analysis, we added kept age and blood concentrations of glucose and low-density lipoproteins on the list of independent parameters and added all ocular parameters which were significantly associated with localized RNFL defects in the univariate analysis. It showed that after stepwise dropping of age (*P* = 0.94), neuroretinal rim area (*P* = 0.88), presence of retinal vein occlusions (*P* = 0.99), ocular perfusion pressure (*P* = 0.62), non-glaucomatous optic nerve damage (*P* = 0.37), blood concentration of glucose (*P* = 0.30), age-related macular degeneration (*P* = 0.32) and optic disc hemorrhages (*P* = 0.22), prevalence of localized RNFL defects were significantly associated with higher mean blood pressure (*P*<0.001), higher concentration of low-density lipoproteins (*P* = 0.01), higher prevalence of glaucomatous optic neuropathy (*P*<0.001) and of diabetic retinopathy (*P* = 0.002), and lower total visibility of the RNFL (*P*<0.001) (Table 2).

**Table 2:** Associations (Multivariate Analysis) between the Prevalence of Localized Retinal Nerve Fiber Layer Defects and Ocular and systemic Parameters in the Beijing Eye Study 2006.

Parameter	*P*-Value	Regression Coefficient	Odds Ratio	95% Confidence Interval
Low-Density Lipoprotein Blood Concentration (mmol/L)	0.01	0.35	1.42	1.08, 1.85
Presence of Diabetic Retinopathy	0.002	1.16	3.20	1.53, 6.67
Presence Glaucomatous Optic Neuropathy	<0.001	3.85	46.8	19.4, 113
Mean Arterial Blood Pressure (mmHg)	<0.001	0.06	1.07	1.03, 1.10
Total Retinal Nerve Fiber Layer Visibility Score (0–68)	<0.001	−0.09	0.92	0.88, 0.96

Retinal vessel diameter measurements were available for a sample of 1263 subjects with a mean age of 59.6±7.7 years and a mean refractive error of 0.07±1.44 diopters with no significant difference in age (*P* = 0.98) and refractive error (*P* = 0.09) to remaining study participants without retinal vessel diameter measurements. The sequence of the fundus sectors (temporal inferior region, temporal superior region, nasal superior region and nasal inferior region) with respect to the RNFL visibility was paralleled by the sequence of the fundus sectors with respect to the retinal arterial diameter which decreased significantly (*P*<0.001) from the temporal inferior region (0.116±0.016 mm) to the temporal superior region (0.110±0.014 mm) to the nasal superior region (0.096±0.013 mm) and finally to the nasal inferior region (0.093±0.012 mm). In a similar manner, the retinal vein diameter decreased (temporal inferior region: 0.153±0.020 mm; temporal superior region: 0.145±0.019 mm; nasal superior region: 0.113±0.017 mm; nasal inferior region: 0.111±0.017 mm). Consequently, the RNFL visibility score in the four sectors was significantly correlated with the diameter of the retinal arteries and veins in the respective fundus regions. The correlation coefficients were usually higher for the parameters in the temporal inferior region and the temporal superior region than for the parameter of the two nasal regions.

## Discussion

In our population-based study, prevalence of localized RNFL defects (mean: 3.7±0.45%) was related with higher blood pressure, higher concentration of low-density lipoproteins, higher prevalence of glaucomatous optic neuropathy and diabetic retinopathy, and lower total visibility of the RNFL. Overall, total RNFL visibility was significantly (*P*<0.001) the highest in the temporal inferior region, followed by the temporal superior region, the nasal superior region, the nasal inferior region, the superior region, the inferior region, the temporal region and finally the nasal region. The total RNFL visibility decreased with older age, male gender, dyslipidemia, higher fasting blood concentration of glucose, myopic refractive error, larger optic disc, and presence of glaucomatous or non-glaucomatous optic neuropathy.

The findings of our study agree with previous hospital-based investigations in which the same sequence of fundus sectors with respect to the RNF visibility was found, and which also reported on the spatial association between the sectorial RNFL visibility and the sectorial retinal vessel diameter [13], [14]. This sequence of fundus sectors with respect to the best visibility of the RNFL and the widest retinal vessel diameters correspond to the shape of the neuroretinal rim following the so called ISNT (Inferior-Superior-Nasal-Temporal)-rule in normal eyes, the morphology of the lamina cribrosa with the largest pores and the largest total pore area in the inferior disc region and the superior disc region, and the position of the foveola usually about 0.5 mm inferior to the horizontal optic disc axis [12], [14], [15]. The finding of a sectorial relationship between a decreasing retinal arterial diameter a decreasing sectorial RNFL visibility within the same sector of the same eye suggests this association was due to local intraocular reasons and that it was not mainly due to systemic factors such as arterial hypertension. The finding of a decreasing RNFL visibility with older age corresponded to histomorphometric findings of an age-related loss of optic nerve fibers of about 0.3% per year of life [16]. The result that a lower RNFL visibility score was associated with dyslipidemia is in agreement with studies which showed an association between dyslipidemia and an increased prevalence and incidence of cerebrovascular infarcts. In a similar manner, the association between a decreasing RNFL visibility and higher blood glucose concentrations related to the loss of retinal nerve fibers in eyes with diabetic retinopathy. Since the parameter of blood glucose concentration as compared to the prevalence of diabetic retinopathy showed a better correlation with a decreasing RNFL visibility in the multivariate analysis, one may assume that the loss of RNFL may start before the ophthalmoscopic characteristics of diabetic retinopathy become visible. The association between a higher RNFL visibility and hyperopia and with a smaller optic disc may be explained by a relative crowding of the retinal nerve fibers in hyperopic eyes or eyes with small optic discs, in some eyes leading to the appearance of a pseudopapilledema [16]. Finally, the correlation between a decreasing RNFL visibility and glaucomatous or non-glaucomatous optic nerve damage simply reflected the optic nerve damage in these conditions [3]–[5].

The findings on localized RNFL defects in our study also agreed with the results of previous investigations. The association between localized RNFL defects and elevated arterial blood pressure was recently reported in a hospital-based study which showed that localized RNFL defects, in addition to retinal microvascular abnormalities such as focal and generalized arteriolar narrowing, were significantly associated with the presence and with increasing grades of arterial hypertension. In that study, the correlation coefficients were even higher for the association between localized RNFL defects and arterial hypertension than for the associations between microvascular abnormalities and arterial hypertension [17]. It shows the importance of the ophthalmoscopic examination for localized RNFLDs in patients with arterial hypertension and for the grading of arterial hypertension. One of the reasons for the occurrence of localized RNFL defects may be the development of retinal cotton-wool spots as micro-infarcts in the RNFL in patients with hypertensive retinopathy. In a similar manner, prevalence of localized RNFL defects was significantly with diabetic retinopathy in our study. It again may reflect the previous development of retinal cotton-wool spots, which disappeared after about 2 months and leaving back a localized RNFL defect which remains visible as long as the surrounding RNFL tissue is thick enough to result in a sufficient spatial contrast between the depth of the localized RNFL defect and the height of the surrounding tissue. The association between the localized RNFL defects and glaucomatous optic neuropathy has been described in many previous hospital based studies [3]–[8]. Correspondingly, localized RNFL defects were also associated with the prevalence of optic disc hemorrhages in the univariate analysis in our study. Disc hemorrhages are usually spatially correlated with localized RNFL defects in glaucomatous eyes [18]. In the multivariate analysis after adjusting for the presence of glaucomatous optic neuropathy, the association between localized RNFL defects and disc hemorrhages was no longer significant in our study.

Potential limitations of our study are that first, as for any prevalence study nonparticipation or exclusion of subjects may have led to a confounding effect. We can therefore not exclude the possibility that the exclusion of subjects with opacities in their optic media may have influenced the results. The same accounts however, for the routine clinical examination, so that the results and conclusions of our study may be transferrable into the daily clinical situation. Second, the assessment of the RNFL visibility and of localized RNFL defects is subjective and thus dependent on the examiner. In our study, the main examiner was well trained and controlled by a panel of examiners. In the normal clinical situation, however, experience and the examination skills varies between routine examiners. Third, a multivariate analysis with so many parameters assessed as in the present study with a sample size of 96 subjects (with localized RNFL defects) always runs the risk of an overestimation of the statistical significance of associations and differences and has a marginal statistic power even it is significant. If a Bonferoni correction was applied for adjusting for performing multiple comparisons, most of the *P*-values were however still small than 0.05. Fourth, a lower RNFL visibility does not necessarily allow drawing the conclusion that the RNFL has decreased. Other reasons may play an important role such as an increased opacity of the lens in elder subjects, in particular nuclear cataract. The better correlation of blood glucose concentration compared to diabetic retinopathy prevalence with a decreasing RNFL visibility may thus be caused by an increased opacity of the lens and may not necessarily be due to a decreased RNFL thickness. The change of birefringence of the RNFL caused by different reasons may also be one of the causes for a different visibility of the RNFL. Strength of our study was that it was the first population-based investigation which assessed the RNFL visibility, the occurrence of its localized defects and associated factors and diseases.

In conclusion, in an elderly Chinese population aged 45+years, RNFL visibility was highest in the temporal inferior sector, followed by the temporal superior sector, the nasal superior sector, and the nasal inferior sector. This sequence of fundus sectors was in agreement with the sequence of fundus sectors with respect to the diameter of the retinal vessels. A decreased RNFL visibility was associated with older age, male gender, dyslipidemia, hyperglycemia, myopia, larger optic disc, and glaucomatous or non-glaucomatous optic neuropathy. Prevalence of localized RNFL defects (mean: 3.7±0.45%) was correlated mainly with higher blood pressure, higher concentration of low-density lipoproteins, glaucomatous optic neuropathy and diabetic retinopathy. These data are helpful for the routine ophthalmoscopic examination of the RNFL.
